# Operationalizing systems thinking approach to sustain public health rehabilitation programs: a rapid review and strategic synthesis

**DOI:** 10.3389/fresc.2025.1633596

**Published:** 2025-09-24

**Authors:** Zanib Nafees, Mahmoud AboAlfa, Mohammed Alkhaldi

**Affiliations:** ^1^ School of Physical & Occupational Therapy, Faculty of Medicine, McGill University, Montreal, Canada; ^2^ Faculty of Physical Therapy, Cairo University, Cairo, Egypt; ^3^ School of Health Sciences and Psychology, Department of Public Health, Canadian University Dubai, Dubai, United Arab Emirates; ^4^ Nuffield Department of Medicine, Center for Tropical Medicine and Global Health, University of Oxford, Oxford, United Kingdom; ^5^ The Global Health Network, Middle East and North Africa Network (TGHN MENA), Dubai, United Arab Emirates

**Keywords:** systems thinking, sustainability, rehabilitation, public health, rapid review, low- and middle-income countries

## Abstract

**Background:**

Public Health Rehabilitation Programs (PHRPs) are essential to achieving universal health coverage and disability-inclusive health systems. Despite their importance, sustainability is threatened by demographic pressures, funding variability, and weak system integration. Systems Thinking (ST) provides a structured paradigm to address complexity, identify key leverage points, and embed adaptive capabilities for longer-term program survival. Our aim was to summarise global applications of ST in PHRPs and identify mechanisms that most effectively contribute to sustainability.

**Methods:**

We conducted a rapid review of peer-reviewed literature and global case studies published between 2010 and 2025. The short timeframe was intentionally selected to provide timely, policy-relevant insights while laying the groundwork for more extensive future reviews. Searches in PubMed, Scopus, and WHO repositories identified studies applying ST to sustain PHRPs. Data were thematically synthesized using the WHO 10-step ST framework and the Systems Thinking for Health (ST4H) model.

**Results:**

Six studies from six countries were included. Three mechanisms emerged: (1) Feedback Loops & Adaptive Learning, (2) Stakeholder Engagement & Systems Mapping, and (3) Strategic Leverage Points. Examples from diverse contexts, especially low- and middle-income countries such as Brazil, India, South Africa, and Jordan, demonstrated improved service integration, resilience, and reach.

**Conclusion:**

ST offers a robust framework for addressing persistent sustainability challenges in PHRPs. Embedding ST early in program design, supported by cross-sector engagement, systems literacy, and strong governance, enhances adaptability, equity, and efficiency. This rapid review provides actionable evidence for policymakers and practitioners, while also underscoring the need for context-specific sustainability metrics and broader scoping or systematic reviews to deepen and expand the evidence base.

## Highlights

• PHRPs face sustainability risks that require systemic, not just programmatic, solutions.
• This review demonstrates that targeted ST mechanisms yield measurable sustainability benefits.
• Provides policymakers with a clear roadmap to integrate ST into rehabilitation strategies in both high- and low-resource contexts.

## 1 Introduction

Rehabilitation is essential for functional recovery and participation, helping reduce disability-related inequities. Public Health Rehabilitation Programs (PHRPs) are central to achieving universal health coverage, yet their sustainability is frequently undermined by shifting policies, inconsistent funding, and growing service demands (1–3).

In this review, we define PHRPs as a set of inclusive services and interventions aimed at optimizing functioning and reducing disability in individuals affected by illness, injury, or aging, while considering their environmental and social contexts. PHRPs integrate rehabilitation as an essential component of public health systems, alongside health promotion, prevention, and protection, to ensure equitable access for all who need it. They encompass services delivered in inpatient and outpatient hospitals, physiotherapy and occupational therapy clinics, and community-based environments such as homes, schools, and workplaces. The core approach of PHRPs is to embed rehabilitation within the broader public health framework, empowering individuals to regain and maintain their ability to participate fully in daily life, work, and education.

Sustainability is a cornerstone of effective health systems, ensuring that programs continue to deliver equitable, high-quality care amid pressures from aging populations, chronic conditions, and climate-related health challenges. In PHRPs, however, fragmented service delivery, unstable funding models, policy discontinuity, and increasing complexity threaten long-term viability (3, 4). Low- and middle-income countries (LMICs) face resource constraints and workforce shortages, while high-income countries struggle with siloed models of care and shifting political priorities (3, 4). Despite global commitments to universal health coverage, rehabilitation remains insufficiently integrated into health systems in ways that are durable, equitable, and responsive (5–7). These gaps highlight the need for innovative approaches (8, 9).

Systems Thinking (ST) offers such an approach. By illuminating interdependencies, feedback loops, and leverage points, ST enables adaptive planning, stronger integration, and long-term resilience (9). The World Health Organization (WHO) advocates for ST through its *Systems Thinking for Health Systems Strengthening* report and the more recent ST4H framework, emphasizing feedback recognition, interdependency mapping, and context-sensitive leverage (10).

To inform ongoing global rehabilitation policy dialogues in 2025, we conducted a rapid review. Rapid reviews provide timely, policy-relevant syntheses suited to dynamic health domains like rehabilitation, where emerging evidence must be quickly translated into action (11). While narrower than scoping or systematic reviews, they offer a practical evidence base that can serve as a foundation for more extensive inquiries.

This review applies the WHO 10-step ST framework and the ST4H model to examine how ST has been operationalized to sustain PHRPs across diverse contexts, including Canada, the UK, and low- and middle-income countries. Our goal is to provide a concise, actionable synthesis for policymakers and practitioners, while laying the groundwork for future comprehensive reviews.

## 2 Methodology

### 2.1 Study design and rationale

We conducted a rapid review to synthesize evidence on the application of ST to sustain PHRPs. This rapid review design was used to provide timely, policy-relevant insights in alignment with ongoing global rehabilitation policy dialogues in 2025. This approach follows WHO and Cochrane Rapid Review guidance, which supports pragmatic adaptations to balance timeliness with methodological rigor. Rapid reviews are well suited to contexts where decision-makers require immediate evidence and have been shown to yield findings comparable to full systematic reviews when methods are transparent. This review is intended as a foundation for future, more comprehensive scoping or systematic reviews.

### 2.2 Search strategy

We searched PubMed, Scopus, and the WHO Institutional Repository for Information Sharing (IRIS) for literature published between 2010 and 2025. Search terms combined concepts related to systems thinking, rehabilitation, and sustainability using Boolean operators (AND/OR).

Example search string: (“systems thinking”) AND (“rehabilitation” OR “rehabilitation services” OR “public health”) AND (“sustainability” OR “adaptive capacity”).

The full search approach, including all keywords and limits, is provided in Supplementary Appendix 1. In addition, we hand-searched reference lists of included studies and targeted relevant reports from the WHO and Organisation for Economic Co-operation and Development (OECD) (12). Searches were restricted to English-language publications.

### 2.3 Eligibility criteria

Inclusion criteria:

• Peer-reviewed articles, policy reports, or case studies applying ST to PHRPs
• Explicit reference to sustainability, long-term outcomes, or health system integration
• Publications between 2000 and 2024
• Any geographic or economic setting
• Publications written in English

Exclusion criteria:

• Studies/publications without focus on, or providing low evidence level, or unrelated to systems thinking, sustainability, rehabilitation, or health systems
• Purely theoretical or conceptual papers without application examples
• Publications not written in English (since the majority of current research applying Systems Thinking in rehabilitation is published in English, and additional evidence from non-English sources remains limited)
• Publications without a focus on sustainability

### 2.4 Study selection and data extraction

Two reviewers (ZN, MAA) independently screened titles/abstracts and assessed full texts for eligibility. Discrepancies were resolved by consensus or adjudication from a third reviewer (MA).

Data were extracted using a standardized form, capturing:

• bibliographic details
• country/region
• study design
• ST mechanisms applied
• sustainability outcomes
• barriers and enablers

### 2.5 Data synthesis

Findings were synthesized thematically, guided by the WHO 10-step Systems Thinking framework and the ST4H model. Three mechanisms; feedback loops, stakeholder engagement, and leverage points, emerged as dominant organizing categories. Case studies were purposively selected to maximize geographic diversity and provide detailed insights into sustainability outcomes.

### 2.6 Quality appraisal

Given the rapid review design and time constraints, we did not conduct a formal quality appraisal using standardized tools such as the Mixed Methods Appraisal Tool (MMAT), which is commonly employed in systematic reviews. Instead, methodological rigor was ensured through a structured screening and synthesis process: two reviewers (ZN and MAA) independently screened and extracted critical data, while a third reviewer (MA) provided quality oversight and synthesis. This approach allowed us to prioritize studies and reports with clear descriptions of ST applications to rehabilitation and sustainability outcomes, while maintaining transparency and reliability despite the rapid review constraints.

### 2.7 Ethics statement

As this review synthesized published literature only, ethical approval was not required.

## 3 Results

### 3.1 Study characteristics

The rapid review identified relevant studies and reports from a variety of sources, including peer-reviewed journals, WHO technical documents, and case studies from national and regional rehabilitation programs. While the search was not intended to be exhaustive, the included publications represented diverse geographic and economic contexts, with examples from both high-income countries (HICs) such as Canada, the UK, and Australia, and LMICs such as Brazil, India, South Africa, and Jordan.

The body of evidence included:

• Implementation case studies of national or regional rehabilitation initiatives;
• Policy analyses examining integration of rehabilitation into broader health systems;
• Qualitative and mixed-methods evaluations focusing on stakeholder perspectives, governance, and operational processes.

Across settings, the studies consistently addressed sustainability in terms of long-term service delivery, integration into health systems, and adaptability to changing needs. Most explicitly described one or more ST mechanisms; feedback loops, stakeholder engagement, and/or leverage points, either as formal frameworks or embedded within practice.

We found a predominance of mixed-methods and qualitative designs, with cases spanning primary care, community-based rehab, and policy reform initiatives. The geographical spread covered all WHO regions, with half originating from LMICs-critical given their unique sustainability constraints Tables 1, 2.

**Table 1:** Study characteristics summary.

Country	Study type	ST Mechanism applied	Level of health system	Outcome focus
Canada	Case Study	Feedback loops	Clinical & Policy	Reduced discharge delays
Brazil	Mixed Methods	Feedback loops	Clinical	Improved patient outcomes, PROM use
South Africa	Qualitative Implementation	Stakeholder engagement	Planning	Resource realignment
UK	Policy Analysis	Feedback loops	National Policy	Reduced regional access disparities
Jordan	Case Study	Stakeholder engagement	Cross-sectoral	Increased coverage, better coordination
India	NGO Operational Case Study	Leverage points	Clinical	Increased service reach

**Table 2:** Barriers and enablers of ST integration in PHRPs.

Category	Barrier	Enabler
Capacity	Low systems literacy among implementers	Training and capacity-building initiatives
Technical	Complex tools and lack of contextual adaptation	Use of simplified, localized ST models
Coordination	Fragmented inter-organizational relationships	Cross-sector collaboration and joint governance
Infrastructure	Weak or absent data systems	Real-time dashboards and feedback loops
Leadership	Lack of long-term policy commitment	Leadership buy-in and early integration in program design

### 3.2 Thematic findings by ST mechanism

#### 3.2.1 Feedback loops and adaptive learning

Feedback loops were commonly used to monitor performance, identify emerging challenges, and inform program adjustments. Examples included integrating patient-reported outcome measures (PROMs) into routine practice, establishing real-time performance dashboards, and using iterative policy review cycles. In several cases, these mechanisms supported rapid responses to service bottlenecks and improved alignment of resources with local needs.

Feedback loops enabled continuous learning, adaptive program modifications, and rapid course correction through real-time monitoring frameworks.

#### 3.2.2 Stakeholder engagement and systems mapping

Stakeholder engagement was identified as both a process and an outcome of ST-informed interventions. Case studies reported co-design workshops, intersectoral planning committees, and participatory systems mapping as strategies to improve coordination and resource allocation. In multiple settings, this approach revealed duplication of services and gaps in referral pathways, leading to targeted solutions and improved service continuity.

#### 3.2.3 Leverage points for strategic and operational impact

Several initiatives identified specific leverage points, small changes that produced outsized effects on system performance. Examples included adjusting referral protocols to reduce waiting times, revising workforce training targets to address shortages, and optimizing travel routes for community-based providers. These targeted changes often required minimal additional resources but yielded significant improvements in service reach and efficiency.

### 3.3 Barriers and enablers of sustainability

Reported barriers included:

• Limited understanding of ST concepts among practitioners and policymakers;
• Complexity of ST tools and lack of contextual adaptation;
• Weak data infrastructure and fragmented monitoring systems;
• Poor inter-organizational communication.

Reported enablers included:

• Strong leadership and policy-level commitment;
• Early integration of ST tools into program design;
• Cross-sector partnerships and shared accountability frameworks;
• Use of real-time data to support decision-making.

## 4 Discussion

This rapid review synthesised evidence on how ST can be applied to improve the sustainability of PHRPs across a range of settings. The review highlights three mechanisms; feedback loops, stakeholder engagement, and leverage points, as recurring features of successful, sustainable rehabilitation initiatives. These mechanisms function not in isolation but as interconnected processes that help programs adapt to complex and evolving health system demands.

### 4.1 Interpretation of findings

The findings confirm that feedback loops are central to sustaining quality and responsiveness in rehabilitation systems. Integrating real-time data into decision-making allows programmes to detect inefficiencies early and adjust resources accordingly, a finding consistent with prior WHO guidance on adaptive governance. Similarly, stakeholder engagement and participatory systems mapping emerged as critical for aligning policies, resources, and services. These processes not only improved coordination but also built trust between sectors and across governance levels, which is essential for long-term sustainability.

Leverage points, small but strategic system changes, were shown to yield significant improvements in access, efficiency, and retention. This aligns with Donella Meadows' original framing of leverage in complex systems and reinforces the idea that well-targeted interventions can achieve disproportionate impact without major new investments.

### 4.2 Comparison with existing literature

While ST frameworks have been widely promoted in health systems strengthening, their application to rehabilitation remains under-documented. This review's examples from diverse contexts, from rural physiotherapy networks in India to integrated stroke systems in Canada, illustrate the versatility of ST across income levels and service delivery models. These findings are consistent with the World Health Organization's *Systems Thinking for Health Systems Strengthening* report, which emphasises context-sensitive application and capacity building as prerequisites for success.

### 4.3 Strengths and limitations

A key strength of this review is its focus on synthesising both peer-reviewed and policy-oriented literature, offering a practice-informed view of how ST can support rehabilitation sustainability. The inclusion of examples from both HICs and LMICs provides a comparative perspective and broadens relevance.

However, the review's scope is limited by its *rapid* design. The 3-month review period (January–March 2025) was intentionally chosen to align with concurrent policy dialogues on rehabilitation integration, enabling timely contributions to those processes. This necessarily meant prioritising relevance over exhaustiveness. Grey literature searching was targeted but not comprehensive, and database searches were restricted to English-language publications. Consequently, relevant non-English or less-accessible studies may have been missed. Furthermore, the descriptive nature of included studies limits the ability to assess causal relationships between ST application and sustainability outcomes.

### 4.4 Policy and practice implications

The evidence suggests that embedding ST mechanisms early in programme design, supported by leadership commitment and robust data infrastructure, can significantly enhance sustainability. Policymakers should consider building ST capacity within rehabilitation teams, integrating participatory mapping into planning cycles, and identifying potential leverage points during programme inception. In resource-constrained settings, low-cost ST applications, such as optimising referral routes or revising discharge protocols, offer feasible entry points for system improvement.

In HICs, ST is best applied to fine-tune system integration and address service inequities; in LMICs, pragmatic leverage points coupled with basic feedback systems can yield rapid, scalable improvements without heavy infrastructure investment.

### 4.5 Future research

Further research is needed to evaluate the long-term impact of ST-informed interventions in rehabilitation, particularly through comparative or longitudinal studies. Expanding the evidence base to include more LMIC contexts and incorporating patient and community perspectives will strengthen the understanding of how ST can be adapted to diverse environments. A subsequent scoping or systematic review could build on this rapid synthesis to provide a more exhaustive mapping of the literature and identify research gaps.

## 5 Conclusion

This rapid review demonstrates that ST offers a practical and adaptable framework for enhancing the sustainability of PHRPs. Across varied geographic and economic settings, three mechanisms; feedback loops, stakeholder engagement, and leverage points, were consistently associated with improved coordination, adaptability, and long-term viability of rehabilitation services.

By intentionally applying these mechanisms, programmes can respond more effectively to shifting demands, align resources with needs, and build resilience within complex health systems. While the review's short timeframe limited the breadth of literature captured, this synthesis provides a timely foundation for policymakers, practitioners, and researchers seeking to integrate ST principles into rehabilitation planning and evaluation.

Future work should expand the evidence base through more comprehensive scoping and systematic reviews, incorporate community and patient perspectives, and evaluate the sustained impact of ST-driven interventions over time. Strengthening systems literacy and fostering cross-sector collaboration will be key to ensuring that rehabilitation services remain accessible, equitable, and effective in the face of evolving health system challenges.

Systems Thinking is not an abstract theory but a practical lens and toolkit for achieving sustainable rehabilitation systems. By institutionalising feedback loops, embedding structured stakeholder engagement, and exploiting leverage points, PHRPs can become more resilient, equitable, and efficient in the face of future shocks.
