# Supplementary material for: Operationalizing systems thinking approach to sustain public health rehabilitation programs: a rapid review and strategic synthesis
Source: Front Rehabil Sci. 2025 Sep 24;6:1633596. doi: 10.3389/fresc.2025.1633596 (PMC12504261; doi:10.3389/fresc.2025.1633596)
Supplement: Supplementary file 1 [file Supplementaryfile1.docx]

**Supplementary Appendix 1 — Search Strategy**

**Databases searched:**

- PubMed
- Scopus
- WHO Institutional Repository for Information Sharing (IRIS)

**Search dates:**

- Between January 1 and March 31, 2025

**Language restriction:**

- English only

**Time frame of publications considered:**

- Publications from 2010 to 2025

**Search approach:**

- Keywords related to Systems Thinking (“systems thinking,” “systems approach,” “systems mapping,” “complex systems,” “causal loop”)
- Keywords related to Rehabilitation (“rehabilitation services,” “rehabilitation programs,” “rehabilitation systems”)
- Keywords related to Sustainability (“sustainability,” “long-term outcomes,” “program durability,” “service continuity”)
- Boolean operators (AND/OR) were used to combine the three main concepts.
- Searches were conducted directly in the database search bars without advanced filters beyond language and publication date.

**Additional sources:**

- Reference lists of included articles were reviewed for further studies.
- Relevant WHO and OECD reports were located through targeted keyword searches within their online portals.

**Note on rapid review methodology:**This search strategy was designed for a *rapid review* to deliver timely, policy-relevant findings. The approach prioritized efficiency and relevance over exhaustive retrieval, acknowledging that future scoping or systematic reviews could expand and deepen the evidence base.
